# MOFormer: navigating the antimicrobial peptide design space with Pareto-based multi-objective transformer

**DOI:** 10.1093/bib/bbaf376

**Published:** 2025-11-05

**Authors:** Li Wang, Xiangzheng Fu, Jiahao Yang, Xinyi Zhang, Xiucai Ye, Tetsuya Sakurai, Xiangxiang Zeng, Yiping Liu

**Affiliations:** Department of Computer Science, University of Tsukuba, 1-1-1 Tennodai, Tsukuba, Ibaraki 305-8577, Japan; School of Chinese Medicine, Hong Kong Baptist University, 7 Baptist University Road, Kowloon Tong, Kowloon, Hong Kong 999077, China; College of Computer Science and Electronic Engineering, Hunan University, Lushan South Road, Yuelu District, Changsha, Hunan 410082, China; College of Computer Science and Electronic Engineering, Hunan University, Lushan South Road, Yuelu District, Changsha, Hunan 410082, China; Department of Computer Science, University of Tsukuba, 1-1-1 Tennodai, Tsukuba, Ibaraki 305-8577, Japan; Department of Computer Science, University of Tsukuba, 1-1-1 Tennodai, Tsukuba, Ibaraki 305-8577, Japan; College of Computer Science and Electronic Engineering, Hunan University, Lushan South Road, Yuelu District, Changsha, Hunan 410082, China; College of Computer Science and Electronic Engineering, Hunan University, Lushan South Road, Yuelu District, Changsha, Hunan 410082, China

**Keywords:** antimicrobial peptide, deep generative networks, multi-objective trade-off, Transformer, Pareto front

## Abstract

Antimicrobial peptide (AMP) design through deep learning holds the potential to revolutionize antibiotic development. Despite recent progress in AMP generation, designing peptide antibiotics with multiple optimal properties remains a significant challenge. We present MOFormer, an advanced multi-objective AMP design pipeline capable of optimizing multiple AMP properties simultaneously. By leveraging a conditional Transformer, the model refines the AMP sequence-property landscape for efficient multi-objective generation. It also incorporates regularization techniques to maintain a highly structured space, enabling the sampling of precise and desirable candidates. Comparative analyses reveal that MOFormer achieves the optimal hypervolume in the multi-objective space, surpassing advanced methods in simultaneously maximizing antimicrobial activity (minimum inhibitory concentration) and minimizing hemolysis and toxicity, thereby yielding the most promising and desirable set of candidate peptides. When extended to a tri-objective scenario, MOFormer continues to exhibit remarkable optimization performance. Finally, we execute a hierarchical and rapid ranking of generated candidates based on Pareto fronts. We conducted a comprehensive validation of the physicochemical properties and target attributes of the candidates, while AlphaFold structure predictions revealed notably reliable predicted local distance difference test scores ranging from 70% to 87%. Our findings suggest that MOFormer holds potential to accelerate the discovery of efficacious peptide antibiotics by optimizing multi-objective trade-offs.

## Introduction

As the development of new antibiotics significantly lags behind market demands, antimicrobial peptides (AMPs) have emerged as promising alternatives to conventional antibiotics, offering potent mechanisms against antimicrobial resistance [1–3]. AMPs are characteristically amphipathic, with cationic amino acids constituting the peptide’s hydrophilic aspect and hydrophobic residues predominating the opposing side. This structural amphipathicity, coupled with their substantial charge, enables AMPs to penetrate and compromise the integrity of negatively charged bacterial cell membranes [4]. Although AMPs rely on cationic amino acids and strong hydrophobicity to exert effective activity against pathogens, these physicochemical properties may also lead to significant hemolysis (HEMO) and toxicity (TOXI), posing potential risks to host cells and presenting challenges for clinical application.

A variety of deep generative model architectures have been explored to enhance AMP discovery, with significant attention devoted to foundational models including variational autoencoders (VAEs) [5–12], generative adversarial networks (GANs) [13–17], recurrent neural networks (RNNs) [18, 19], and graph neural networks [20]. Muller *et al*. [19] employed RNNs with long short-term memory (LSTM) units for the pattern recognition of helical AMPs, particularly focusing on linear cationic peptides that form amphipathic helices. This approach aimed to closely model the fundamental attribute of amphipathicity, which is closely associated with antimicrobial activity. Szymczak *et al*. [12] introduced HydrAMP, a conditional VAE designed to learn a lower dimensional, continuous representation of peptides and successfully generated highly active analogs showcasing potent antimicrobial properties. Zhang *et al*. [17] developed the specialized PepGAN architecture, which optimally balances the identification of active peptides while avoiding non-active variants. Additionally, Liu *et al*. [21] implemented an evolutionary multi-objective algorithm that effectively harnesses *de novo* AMP generation, with a focus on optimizing for both antimicrobial activity and peptide diversity. These collective efforts underscore the diverse computational approaches employed to model and enhance the design of effective AMPs, highlighting the pivotal role of deep learning in advancing biotechnological innovations.

Despite these encouraging findings, there are still idealism–realism limitations to tackle in the process of producing new AMP with targeted properties. (1) First, discrete sequence data generation, which is more challenging than continuous data generation. The AMP sequence alone is insufficient to probe the direct structural information and properties of the peptide. (2) Subsequently, overemphasizing diversity at the expense of inherent conflicting properties in AMP design contributes to a high clinical attrition rate.

The rapid advancements in natural language processing may provide valuable insights for the future of artificial intelligence-designed peptides, potentially establishing them as the norm. The transformer architecture, foundational to several state-of-the-art models such as BERT [22] and molGPT [23], shows considerable promise in the design of AMPs. The self-attention mechanism of the Transformer allows for capturing long-range relationships in sequence data [24]. This capability is crucial for understanding the functional features of AMPs, which often involve interactions between amino acid residues that are distant in the sequence. Moreover, the continuous interpolatability characterizing the latent space of a Transformer-based generator facilitates directed interpolation or sampling within the sequence space under specific conditions, enhancing the model’s flexibility and efficiency in generating desired AMP properties.

Given the inherent complexities of AMPs, the design of AMPs can thus be framed as a multi-objective optimization problem (MOP) that balances antimicrobial activity [minimum inhibitory concentration (MIC)] and either HEMO or TOXI properties. The ideal trade-offs among these objectives can be defined through Pareto optimality, where a decision-maker requires an approximation to the Pareto front (PF) to select a final preferred solution—candidate AMPs [25]. Most MOP may present many, or even an infinite number of, Pareto optimal vectors, and acquiring a complete PF is typically labor-intensive and often unfeasible. Consequently, many MOP optimizers aim to identify a manageable number of Pareto optimal vectors that are evenly distributed along the PF, serving as effective representatives of the entire front. These representative vectors enable the screening of desired AMPs that possess multifaceted properties.

**Figure 1:**
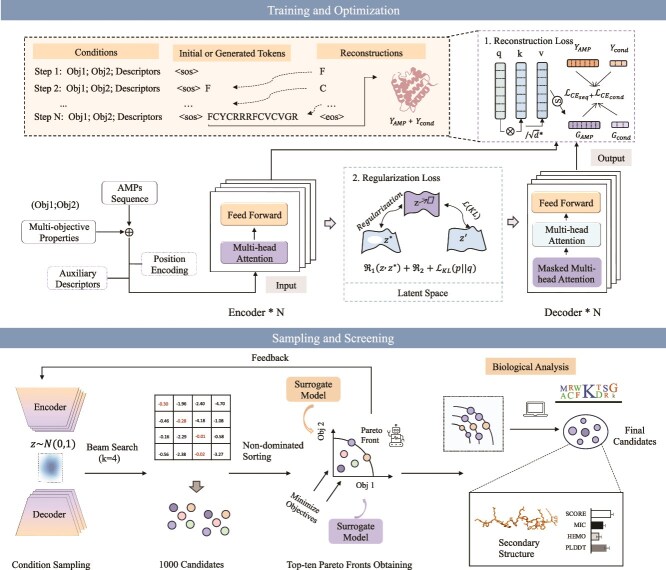
The MOFormer pipeline. The MOFormer pipeline comprises a two-phase framework integrating multi-fusion descriptors, Transformer-based modeling, and hierarchical screening to enable multi-objective antimicrobial peptide generation and optimization.

In this study, we introduced MOFormer, a deep transformer-based framework for designing AMPs, which capitalizes on multi-objective properties and multi-fusion descriptors to generate desirable candidates within a regularized latent space. The architecture of the proposed approach is illustrated in Fig. 1. The contributions of our study are summarized as follows:

(1) For realistic and desirable AMPs, we introduced the Multi-Objective Transformer (MOFormer), embedded within a highly structured latent space, specifically designed to effectively navigate and optimize the trade-offs in conflicting multi-property design for AMPs. Experimental results consistently demonstrate that MOFormer achieves optimal hypervolume (HV) across a broad spectrum of multi-objective spaces, surpassing state-of-the-art methods while exhibiting superior robustness and performance across diverse optimization scenarios.
(2) For robust screening protocol, we introduced a non-dominated sorting algorithm based on multi-objective optimization, which provides a fast decision-making process and a set of trade-off solutions. This algorithm can be combined with a suite of fine-tuned surrogate models (such as MIC predictors) to generate multiple Pareto decision curves, and the solutions on the top-ranked curves are regarded as candidates for more detailed screening to help researchers locate the most potential candidates.
(3) To ensure the validity of the candidates and enhance the MOFormer’s interpretability, we performed multiple sequence alignment and molecular visualization to thoroughly analyze the sequence characteristics and structural functions. Additionally, we visualized the structured latent space and calculated attention scores between amino acids, providing researchers with more detailed and informative references.

## Methods

### Datasets

For our analysis, we compiled training samples consisting of sequences up to 60 amino acids in length, sourced from studies by Witten *et al*. [26], Szymczak *et al*. [12], and Hasan *et al* [27]. After performing multiple sequence alignments and applying a truncation ratio of 0.35 to eliminate redundancy, our final dataset comprised 4096 AMP sequences. These sequences are experimentally verified to have low MIC against E. coli, with values in the range [−1, 4], and demonstrate a HEMO probability below 0.7. This filtering reduces distributional noise, mitigates extreme gradient effects in multi-objective learning, and alleviates class imbalance, which would otherwise compromise convergence and limit the model’s capacity to faithfully capture the trade-offs among multiple antimicrobial objectives.

Additional datasets for training sub-problem models include 4547 AMP samples with antimicrobial activities, 1006 balanced samples for HEMO and non-HEMO activity from Hasan *et al*. [27], and 201 TOXI samples alongside an equal number of non-TOXI controls from Das *et al*. [28]. Note that the toxic annotation here does not depend on a specific type of toxicity, such as HEMO toxicity. Hemolytic activity is typically measured by the concentration at which 50% HEMO(HC50) occurs, with HC50 indicating the peptide concentration that causes lysis of 50% of red blood cells. In contrast, TOXI is assessed using the lethal dose (LD50), defined as the dose required to cause death in 50% of experimental animals, usually expressed in mg/kg. In addition, regression and classification models were subsequently fine-tuned using the large-scale Prot-BERT-BFD model [29].

#### Overview of MOFormer framework

The MOFormer framework consists of two sequential phases: (1) a training and optimization stage and (2) a sampling and screening module for candidate selection. In the first phase, the model is trained under a multi-objective optimization setting, exemplified by the concurrent improvement of MIC and either HEMO or general TOXI. Model inputs include the primary AMP sequences along with their associated property annotations. To augment the representational capacity, auxiliary multi-fusion descriptors—comprising Amino acid composition (AAC) and Composition, Transformation, and Distribution (CTDC) features—are employed. These 59-dimensional vectors are subsequently compressed into three principal components via principal component analysis (PCA), preserving the most informative physicochemical patterns. Concurrently, positional encodings are applied to maintain sequence-order dependencies. The resulting representations are fed into a Transformer-based encoder, which is further constrained using reconstruction loss to regularize the latent space and ensure generation stability. In the second phase, a hierarchical selection strategy is adopted, involving Pareto non-dominated sorting and surrogate modeling based on a fine-tuned Prot-BERT-BFD model. The most promising candidates are then subjected to molecular visualization and interpretative analysis to assess their structural and functional plausibility.

#### Sequence modeling with multi-objective optimization

In complex multi-objective optimization scenarios, the conflicts among different design objectives can be analogized to multiple “players” competing for limited resources—namely, the peptide design space. This game-theoretic perspective has increasingly been adopted in multi-objective peptide design in recent years. Compared with traditional approaches such as weighted-sum methods or evolutionary algorithms, which typically rely on iterative search, conditional generation strategies incorporate desired target properties as explicit control signals, guiding the generative model to satisfy multiple objectives while maintaining structural validity and diversity.

Leveraging insights from Zhao *et al*. [30], we adapted a conditional distribution $p(x|c)$ approach that captures property-specific priors on latent representations in the vanilla transformer architecture (The formulation of Transformer-based sequence modeling is provided in Supplementary Section A). This method facilitates the disentanglement of target properties $c$ from the prior by constructing $p(z|c)$, where changing $c$ adjusts the mean of the resulting Gaussian distribution. This setup allows continuous targets to be encoded directly in the distribution’s mean. For our study, where the target $c$ incorporates multi-objective properties and multi-fusion descriptors for each AMP input $x$, the distribution can be decomposed using the chain rule of probability:


(1) \begin{align*}& p(x|c)=\prod_{i=1}^{n}p(x_{i}|x_{<i},c),\end{align*}

and trained with a loss function that integrates the control code:


(2) \begin{align*}& \mathcal{L}(X)=-\sum_{k=1}^{n}\log p_\theta(x_{i}^{k}|x_{<i}^{k},c^{k}).\end{align*}

We define an MOP for AMPs that considers conflicting properties. Formally, we define the problem as $c(x)=(c_{1}(x),...,c_{j}(x))^{T}$, subject to $x\in \Omega $, where $\Omega $ is the decision space and $c:\Omega \to \mathbb{R}^{j}$ consists of $j$ real-valued objective functions. The attainable objective set is defined as $\{c(x)|x\in \Omega \}$. Since objectives in $c(x)$ often conflict, no single point in $\Omega $ maximizes all objectives simultaneously. For instance, in AMP design, one might aim to maximize antimicrobial activity (measured by MIC) while minimizing HEMO effects:


(3) \begin{align*}& \mathrm{maximize}\,c(x)=\left(c_{mic}(x),c_{hemo}(x)\right)\!.\end{align*}

We utilize multi-objective properties to guide the latent space, in which case the conditional loss can be defined as


(4) \begin{align*}& \mathcal{L}_{\mathrm{ce:{cond}}}(\theta;c,z)=\sum_{k=1}^{n}(c^{k}-g_\theta(z)^{k})^{2},\end{align*}

where c represents the target condition, z is the latent variable generated by the encoder, affected by the input data and the condition c. $g_\theta (z)$ means a function determined by the model parameters, which maps from the latent space to the conditional space and predicts the value of the condition c.

While generative models have shown promise in handling conditional properties of AMPs, determining a comprehensive and effective set of conditions for neural network inputs remains challenging. To address this, we introduce a multi-fusion approach for AMP conditioning that integrates rich information beyond the primary sequence data, encompassing physical, chemical, and structural properties. AAC quantifies the frequency of amino acids within peptide sequences, producing multi-scale feature vectors with rapid transformation capabilities [31]. CTDC offers indirect insights into the structural and physicochemical properties of peptides by analyzing their composition and distribution [32].

By merging these two methods, we create a comprehensive 59D multi-fusion description of AMP functions. Utilizing PCA [33], we reduce the dimensionality of these conditional descriptors, focusing on the most significant variances to capture the core features of the data in three dimensions. These descriptors are further compressed into a final 3D conditional vector. For the training set, the explained variance ratios of PCA1–3 are 21.7%, 15.9%, and 12.5%, summing to about 50.1%. Similarly, for the test set, these values are 20.9%, 16.1%, and 13.1%, totaling 50.2%. While the first three principal components capture roughly half of the variance, this level of dimensionality reduction is sufficient, given the intrinsically dispersed nature of the high-dimensional descriptors, to retain meaningful auxiliary information. The resulting 3D vector is subsequently integrated with multi-objective property constraints to effectively guide the generation of AMPs with desired characteristics. The 59-feature multi-fusion descriptors, PCA loadings, and the resulting 3D control vector have been provided in the Supplementary Materials and are available in our GitHub repository to ensure reproducibility.

#### Regularization strategies

Despite the successes of the transformer architecture, a significant challenge known as posterior collapse often undermines the performance of generative models [34–36]. This issue is generally characterized by the convergence of the Kullback–Leibler (KL) divergence $D_{KL}[q_{\phi }(z|x)||p(z|c)]\to 0$ toward zero for all inputs $x$, typically arising when the decoder is overly powerful. Such convergence suggests that the learned variational distribution closely approximates the prior (e.g. a standard Gaussian distribution), causing the latent representations of different inputs to become indistinguishable in the latent space.

In our approach, we have implemented KL divergence annealing within the loss function to stabilize the gradient flow, complemented by a novel Gaussian dropout regularization strategy. The key equations governing our model’s training are as follows:


(5) \begin{align*} & \mathcal{L}_{KL}(X)=\sum_{k=1}^{n} D_{\mathrm{KL}}(q_\phi(z^{k}|x^{k})||p_\theta(z^{k}|c^{k})), \end{align*}

(6) \begin{align*} & z^{*}=\mu+\alpha*\sigma\Theta\epsilon, \end{align*}

(7) \begin{align*} & \mathcal{R}_{1}(z,z^{*})=\sum_{k=1}^{n}CrossEntropyLoss(z^{k},z^{k^{*}}). \end{align*}

Here, $\alpha $ is a learnable weight parameter that adjusts the Gaussian dropout ratio. Blum *et al*. [37] have further discussed $\alpha $ from a Bayesian regularization perspective, emphasizing its role in minimizing the KL divergence between the posterior distribution and the prior when optimized jointly with the model. We empirically set $\alpha $ at 0.2. Furthermore, we also employ intuitive and effective optimization strategy to encourage the model to aim for low MIC and minimal HEMO potential, we define


(8) \begin{align*}& \mathcal{R}_{2}(z)=\sum_{k=1}^{n}\left\{\left(g_\theta(z)_{mic}^{k}-\varepsilon\right)^{2}+\max\left(0,g_\theta(z)_{hemo}^{k}-\gamma\right)\right\}^{2},\end{align*}

where $\mathbf{\varepsilon }$ signifies the empirically set minimum MIC value, taken as the log value of $-2$. $\gamma \!$, serving as input to the ReLU activation function, is set at 0.5 to act as the threshold. These approaches together provide a nuanced framework for enhancing AMP design through sophisticated modeling of sequence and property relationships, ensuring the integration of both efficacy and safety profiles in the final peptide candidates.

Summarizing, the overall objective function of our model is defined as


(9) \begin{align*}& \mathcal{L}=\mathcal{L}_{CE_{seq}}(X)+\mathcal{L}_{CE_{cond}}(X)+\beta*\mathcal{L}_{KL}(X)+\mathcal{R}_{1}(z,z^{*})+\mathcal{R}_{2}(z),\end{align*}

where $\mathcal{L}_{CE_{seq}}(X)$ and $\mathcal{L}_{CE_{cond}}(X)$ denote the reconstruction loss, $\mathcal{L}_{KL}(X)$ represents the KL divergence, and $\beta $ signifies the weight of KL, which uses the KL annealing algorithm [38] to gradually increase the weight of the KL divergence term to mitigate the posterior collapse problem, $\mathcal{R}_{1}(z,z^{*}) $ and $\mathcal{R}_{2}(z)$ pertains to the regularization strategy.

#### MIC regression and HEMO classification

Despite numerous predictors available for MIC activity, most rely on simple threshold-based classification for activity versus inactivity, with few methods tailored for regression-based prediction. In our work, we understand AMPs as peptides recorded in databases like APD [39], CAMP [40], LAMP [41], and DBAASP [42]. Addressing both MIC activity and HEMO is crucial, as many AMPs are either inactive against E. coli or harmful to host cells. This provides a strong rationale for employing classifiers and regressors as surrogate models. The MIC value regressor is trained on a broad dataset of AMP sequences, while the HEMO classifier is trained on a more selective dataset of sequences known for HEMO properties. Both models employ the pretrained protein sequence model, Prot-BERT-BFD [29], trained on over 2 billion protein fragments. Additional fine-tuning involves multiple fully connected layers and batch normalization, with the final layer’s activation function tailored to perform either regression for MIC values or classification for HEMO properties. Implemented in PyTorch, these models achieve exceptionally low mean squared errors and exceed 95% accuracy on independent validation sets.

Finally, candidate peptides are generated based on the conditional sampling strategy detailed in Section B of the Supplementary Materials. These candidates are subsequently evaluated using fine-tuned large-model-based predictors and classifiers to facilitate non-dominated sorting and iterative feedback optimization.

#### Pareto-based feedback and optimization

To design AMPs with optimal pharmacological properties and enhanced diversity, we employed a Pareto-based feedback optimization framework, integrating multi-objective optimization principles. This approach utilizes a transformer-encoder, continuously refined via a feedback loop that incorporates performance metrics such as MIC and HEMO. Each iteration’s top-10 AMP candidates, ranked by PF, are reincorporated into the training dataset, thus enriching the model with AMPs that exhibit more desirable traits. The top-ranked PFs—especially PF1—comprise non-dominated AMP candidates that exhibit favorable trade-offs, such as low MIC values and low HEMO risk. By focusing the feedback loop on these high-quality candidates, the model is guided toward biologically meaningful and therapeutically relevant regions in the objective space.

The MOP framework applies a non-dominated sorting algorithm to classify AMPs into multiple PFs based on their performance across various objectives. A PF includes solutions that are not inferior to any alternatives and are superior in at least one objective function, representing non-dominated solutions. A solution is deemed Pareto optimal if no alternative solution outperforms it across all objectives simultaneously, i.e. any improvement in one objective would necessarily result in the deterioration of at least one other. This principle embodies the inherent trade-offs central to multi-objective optimization. Hence, the MOP aims to optimize multiple objective functions simultaneously. In a minimization context, for vectors $u$ and $v$, vector $u$ is said to dominate vector $v$ if $u_{i} \geq v_{i}$ for all indices $i \in{1,..., m}$ and $u_{j}> v_{j}$ for at least one index $j \in{1,..., m}$. Within this framework, the non-dominated sorting employs internal and external loops; the former stores solutions in distinct top PFs, and the latter assesses whether a candidate dominates the solution space. The refined optimal PF contains AMPs with advantageous trade-off characteristics, namely lower MIC values and reduced HEMO risk.

In each iteration, the generated AMP candidates are evaluated using objective metrics (e.g. MIC and HEMO) and ranked through non-dominated sorting into PFs. Sequences from the top 10 PFs (PF1–PF10) are then re-encoded and reintegrated into the training set, with their predicted property scores serving as auxiliary supervision. This iterative process progressively guides the model toward pharmacologically favorable regions within the objective space, while simultaneously enhancing both the diversity and overall quality of the generated AMPs. The implementation details of MOFormer are provided in Supplementary Section C.

Overall, the proposed feedback optimization strategy, based on the multi-objective PF, leads to the generation of AMP candidates with desired properties, addressing the need for specific attribute datasets to some extent.

## Results

### MOFormer outperforms existing methods

MOFormer is specifically designed to generate AMPs with multiple properties while adhering to specified conditions. To access the contributions of key components in our proposed framework, we performed an ablation study by training MOFormer under three distinct settings: MOFormer (w/o D), MOFormer (w/o F), and the full version of MOFormer. This design allows us to evaluate the impact of (1) fine-grained peptide descriptors and (2) the feedback loop mechanism on the quality of generated peptides.

MOFormer (w/o D): this variant removes the fine-grained descriptors during training. It serves as a baseline to assess model performance without detailed physicochemical guidance.

MOFormer (w/o F): this version excludes the feedback loop, allowing us to isolate and examine the benefit of iterative refinement based on model predictions.

MOFormer: the complete model integrates both descriptors and the feedback mechanism, facilitating guided generation under specific property constraints and enabling iterative enhancement of multi-objective optimization performance.

The performance of MOFormer was benchmarked against six state-of-the-art methods: LSTM [19], AMP-GAN [43], PepGAN [17], WAE [44], AMPEMO [21], and HMAMP [45].

LSTM utilizes RNNs for peptide sequence generation but lacks explicit mechanisms for controlling multiple objectives, thereby limiting its ability to steer generation toward desired property profiles.

AMP-GAN and PepGAN incorporate generative adversarial frameworks to enhance the realism and diversity of generated sequences. However, their optimization is predominantly focused on antimicrobial activity, without integrated mechanisms for multi-objective control or interpretability.

WAE adopts a latent space modeling approach that improves representation learning, yet it relies on particle swarm optimization, which introduces significant computational overhead and constrains generative diversity.

AMPEMO employs an evolutionary multi-objective optimization framework to explore trade-offs between antimicrobial activity and diversity, achieving broad PF coverage. Nonetheless, its non-differentiable, search-based nature results in limited generative efficiency and adaptability.

HMAMP introduces an HV-based loss into a multi-objective GAN framework, enhancing the quality of generated samples under multiple constraints. Despite these advances, the method inherits training instability from adversarial networks and lacks fine-grained control over specific target properties.

Despite the notable advances by AMPEMO and HMAMP in multi-objective optimization, the exploration of latent space optimization techniques based on multi-objective optimization is still an emerging area that has yet to be fully explored. In contrast, MOFormer combines property-aware conditional generation with a feedback mechanism in a transformer architecture, enabling end-to-end and efficient multi-objective optimization without external search or adversarial training, providing a stable and scalable solution for AMP design.

**Figure 2:**
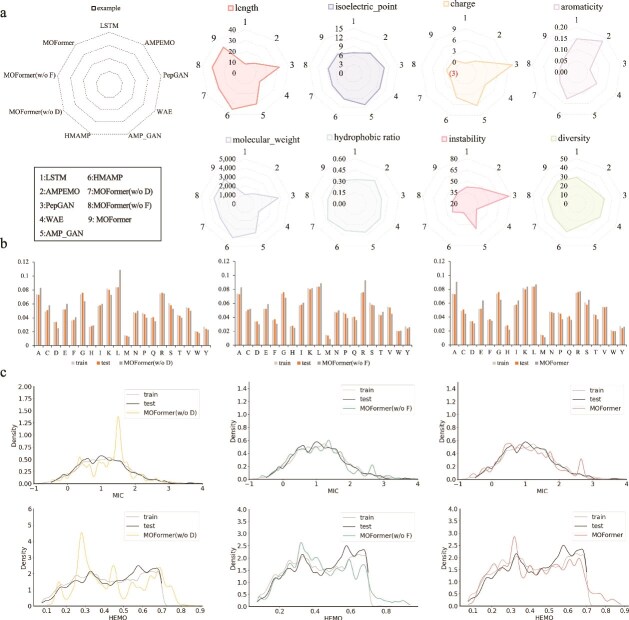
Comparative analysis of AMP generation by MOFormer and other methods. a. Fundamental properties of AMPs generated by MOFormer compared with other state-of-the-art methods, highlighting MOFormer’s enhancements in generating AMPs with optimized properties. b. Amino acid distribution comparison among AMPs generated by MOFormer, MOFormer (w/o D), and MOFormer (w/o F), illustrating the impact of different training modes on sequence diversity. c. Attribution distribution comparison among AMPs generated by MOFormer, MOFormer (w/o D), MOFormer (w/o F), and a benchmark dataset, demonstrating MOFormer’s effectiveness in aligning generated AMP properties with those of known efficacious peptides.

To demonstrate our findings, we focused on MIC and HEMO as primary objective properties. We trained entire model and sampled 1000 AMPs, which were then assessed using both characteristic-related metrics and the multi-objective evaluation metric HV. The characteristic metrics considered include length, isoelectric point, charge, aromaticity, molecular weight, instability, hydrophobic ratio, and diversity, as depicted in Fig. 2(a). It is worth noting that the calculated diversity score for the MOFormer-generated peptide set is 32.691, indicating a high level of sequence-level variation. This result suggests that the model effectively mitigates mode collapse and is capable of exploring a broad and diverse region of the sequence space.

In addition, we employed two complementary evaluation strategies to assess the novelty of the generated sequences relative to the training data. (1) A generated sequence was classified as novel if its Levenshtein distance from every sequence in the training set exceeded a predefined threshold T. The corresponding results are presented in Supplementary Table I. When T = 3, the novelty score reached 0.289, indicating that approximately one-third of the generated peptides are structurally distinct from any sequence in the training set. (2) We also computed the average minimum Levenshtein distance between each generated sequence and its most similar counterpart in the training set. The resulting average minimum distance of 3.672 demonstrates that the generated peptides consistently differ from the training data by more than three sequence edits, thereby highlighting the model’s ability to produce structurally novel sequences without reliance on a fixed threshold.

Our comprehensive evaluation reveals that although MOFormer is not the top performer in every metric, it ranks well within the higher tier of methods compared in terms of overall efficacy. This balance across multiple characteristics underlines MOFormer’s utility in generating AMPs that effectively combat microbes with minimal risk to host cells.

The HV metric [46] is a unary metric used to measure the extent of the objective space covered by an approximation set, requiring a reference point, $\eta $, for calculation. In this study, we set $\eta $ as (2, 0.5), corresponding to our targets for antimicrobial activity $(log(MIC) < 2)$ and HEMO probability $(HEMO < 0.5)$. A higher HV value intuitively indicates that the model generates more Pareto-optimal or near-optimal solutions that effectively balance competing objectives. In contrast to single-objective metrics such as average MIC or HEMO, HV provides a comprehensive evaluation by simultaneously considering three key aspects: the proximity of generated solutions to the PF (convergence), the spread and coverage of solutions across the objective space (diversity), and the number of non-dominated solutions within the set (cardinality). Overall, HV is widely regarded as one of the most informative and robust measures for evaluating model performance in multi-objective AMP design.

For an intuitive comparison of performance across methods, Table 1 summarizes the outcomes of the generation process. These visualizations display the percentage of peptides falling within each attribute range for MIC and HEMO, the percentage of candidate AMPs meeting all attribute criteria, and the HV metric results.

**Table 1:** The comparison of the interval satisfaction proportions of AMPs generated by MoFomer versus other methods, along with a comparison of HV multi-objective metric, aimed toward MIC and HEMO properties

Method	MIC (<2)	HEMO (<0.5)	Combination	HV
LSTM	0.63	0.831	0.498	0.566
AMPEMO	0.82	0.443	0.347	0.439
PepGAN	0.152	0.945	0.143	0.718
WAE	0.841	0.338	0.245	0.591
AMP_GAN	0.984	0.393	0.381	0.720
HMAMP	0.94	0.350	0.305	0.891
MOFormer (w/o D)	0.622	0.898	0.575	0.767
MOFormer (w/o F)	0.622	0.886	0.518	0.930
MOFormer	0.596	0.881	0.502	0.976

While the AMPs generated by our methods (MOFormer, MOFormer w/o D, and MOFormer w/o F) may not always show the highest interval occupancy ratios for MIC and HEMO, their overall performance is significant, particularly in terms of HEMO. This outcome likely stems from our strategy of balancing multiple objectives, rather than prioritizing one property over others. In the combined optimization of MIC and HEMO, MOFormer and its variants outperform other methods, with MOFormer demonstrating a slightly lower performance compared with its variants. This can be attributed to MOFormer’s preference for generating candidates with lower MIC values and reduced HEMO probabilities, whereas MOFormer (w/o D) and MOFormer (w/o F) tend to produce a larger fraction of candidates nearing the optimal threshold.

Notably, among the nine methods evaluated, MOFormer achieves the best HV result, effectively advancing in the direction opposite to the reference point. This suggests that MOFormer most effectively controls the balance between MIC and HEMO, aligning with the overarching goals of this research. In addition, to enhance our understanding of how MOFormer optimizes AMPs with respect to multiple objective properties, we provide a visual illustration of the attribute landscapes and AMP embedding space, as shown in Supplementary Figure S1. We observed that MOFormer adeptly captures intricate distinctions in AMPs, achieving a harmonious and even feature distribution.

### Characteristics of the designed AMPs

To assess the homology of AMP sequences generated by MOFormer with those from the training and testing datasets, we analyzed the AAC distributions, as depicted in Fig. 2(b). Figure 2(c) presents the distribution of MIC and HEMO for MOFormer-generated AMPs compared with the train and test datasets. MOFormer (w/o F) and MOFormer models more closely mirror the distribution of the original dataset compared with MOFormer (w/o D). Notably, MOFormer demonstrates a consistent leftward shift in MIC, indicating an enhancement of antimicrobial efficacy—a result of the optimization processes implemented. In terms of HEMO, both MOFormer (w/o F) and MOFormer show trends toward reduced HEMO activity, with distributions notably shifting leftward compared with MOFormer (w/o D). This trend is more pronounced in relation to MIC. Despite these optimizations, all models produce a small fraction of AMPs with HEMO values above 0.8, reflecting the intrinsic trade-offs encountered during multi-objective optimization and the challenges in balancing diverse properties.

However, as illustrated in Fig. 2c, the density of generated samples within the ultra-low MIC region (MIC<0) and the extremely low HEMO range (0.1–0.2) remains sparse, underscoring the relative rarity of sequences that simultaneously satisfy both stringent criteria. This phenomenon likely arises not only from distributional limitations inherent to the training data, but also from an intrinsic trade-off—where pushing toward more extreme target values inherently constrains and diminishes the achievable sequence diversity.

Significance testing using the T-test further aids in understanding the differences between the samples. As summarized in Supplementary Table II, MOFormer model demonstrates elevated diversity in the MIC property $(P-value <.05)$, yet exhibits diminished diversity in the HEMO property, which predominantly focuses on low-probability events. Overall, there is a pronounced similarity between the training and testing datasets, particularly in the HEMO property. This suggests that the MOFormer model not only effectively assimilates the original data but also preserves a robust diversity. MOFormer (w/o D) shows markedly high diversity in the MIC attribute $(P-value <.0001)$, but exhibits reduced diversity and similarity in the HEMO property. Omitting descriptor information leads to deviations in data generation for certain features compared with the original data. Conversely, MOFormer(w/o F) maintains high similarity across both MIC and HEMO properties, yet the diversity is significantly compromised. The absence of feedback appears to restrict the variability and diversity of the generated data.

### Sequential and mechanistic analyses

**Figure 3:**
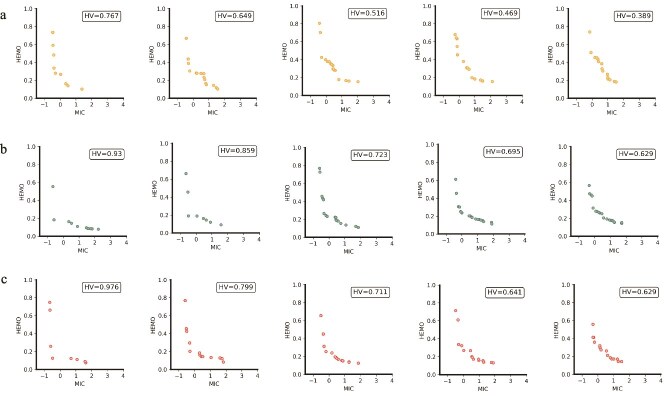
Pareto fronts of MOFormer variants. This figure illustrates the top five PFs for three different configurations of the MOFormer model: a. MOFormer (w/o D), b. MOFormer (w/o F), and c. MOFormer.

To evaluate the 1000 AMP sequences generated by MOFormer, we first identified the top-five PFs through non-dominated sorting. As depicted in Fig. 3, the two objectives to be minimized among these fronts appear to best meet our criteria, demonstrating high antibacterial activity with low HEMO. The MOFormer (w/o D) displays significantly lower HV values on all PFs, underscoring the vital role descriptors play in capturing AMP-related features. Their absence leads to a reduced solution space and lower diversity, highlighting their importance in multi-objective generation. Conversely, MOFormer (w/o F) achieves higher HV values, demonstrating its ability to autonomously generate a diverse set of high-quality solutions. This suggests a robust underlying optimization algorithm, though the lack of feedback may limit the discovery of optimal solutions. The first PF of MOFormer records the highest HV value, indicating the effectiveness of the feedback strategy in boosting top-tier solutions by reincorporating the most promising candidates back into the training set, thus achieving superior optimization results. However, the decrease in HV values in subsequent fronts suggests that focusing too heavily on top solutions could compromise overall diversity.

We selected all points from the top five PFs, comprising a total of 67 candidates, for comprehensive structural and functional analysis. These 67 candidates were further screened using a conventional AMP classifier, a Support Vector Machine, which identified 56 candidates exhibiting optimal antibacterial activity and minimal HEMO potential.

To further investigate the functional characteristics of the 56 candidate sequences, sequence logos were used to analyze amino acid conservation and variability at specific positions. As shown in Supplementary Figure S2(a), conserved residues with high information content (measured in bits) likely contribute to key functional or structural roles in AMP activity. These conserved motifs provide valuable insights for guiding subsequent sequence optimization and experimental validation.

### Structural and interpretable analyses

In the realm of antimicrobial research, short-chain AMPs are distinguished by their compact molecular architecture and straightforward structural configurations, which facilitate their penetration through bacterial membranes and biofilms. Their structural simplicity also renders them less susceptible to enzymatic degradation, enhancing both *in vivo* stability and therapeutic potency. With an optimization goal aimed at maximizing antimicrobial proficiency while minimizing HEMO, we selected five candidates with lengths of 25 or fewer amino acids from the initial pool of 56 for detailed molecular visualization and functional analysis.

**Figure 4:**
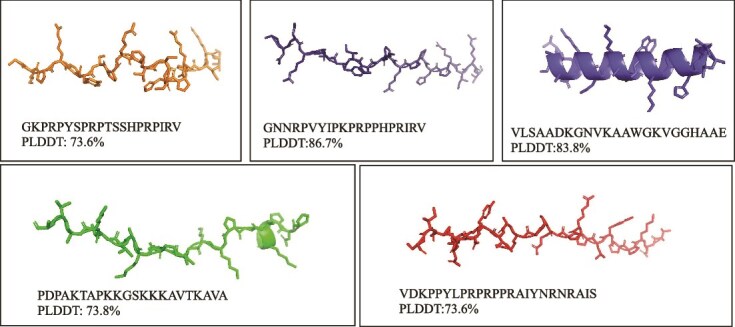
AMP structures derived from molecular visualization for peptides ID1 through ID5, showcasing the molecular conformation linked to their bioactivity.

The structural conformations of candidates ID1 through ID5 were predicted using the AlphaFold platform and visualized with PyMOL. As illustrated in Fig. 4 and summarized in Table 2, ID1, ID2, ID4, and ID5 predominantly adopt random coil structures. Such conformational flexibility is likely to promote dynamic interactions with bacterial membranes of varying lipid compositions, thereby enhancing membrane targeting and antimicrobial activity. Conversely, ID3 exhibits a well-defined alpha-helical structure, a motif commonly associated with membrane insertion and disruption in potent AMPs. Its high predicted local distance difference test (PIDDT) score of 83.8% indicates strong structural stability, which may confer resistance to enzymatic degradation and prolonged *in vivo* persistence. Collectively, these structural characteristics suggest complementary functional advantages: coil-rich peptides (ID1/2/4/5) may offer broad-spectrum adaptability, whereas the helical peptide (ID3) may provide enhanced stability and sustained antimicrobial efficacy. These insights inform candidate prioritization for subsequent experimental validation.

**Table 2:** Detailed properties and attributes of curated AMPs (ID1-ID11)

ID	Sequence	Length	isoelectric_point	Charge	Aromaticity	molecular_weight	Hydrophobic ratio	instability	MIC(log)	HEMO	TOXI
1	GKPRPYSPRPTSSHPRPIRV	20	11.999	4.588	0.05	2285.609	0.1	49.225	0.184	0.24	-
2	GNNRPVYIPKPRPPHPRIRV	20	11.999	4.588	0.05	2363.767	0.2	25.815	-0.364	0.447	-
3	VLSAADKGNVKAAWGKVGGHAAE	23	8.477	0.555	0.043	2236.485	0.434	-16.669	1.24	0.157	-
4	PDPAKTAPKKGSKKKAVTKAVA	22	10.4	5.884	0	2221.642	0.318	15.909	0.557	0.195	-
5	VDKPPYLPRPRPPRAIYNRNRAIS	24	11.451	4.515	0.083	2847.283	0.25	32.483	0.14	0.298	-
6	KWKFKKIPKFLHLAKKF	17	10.778	6.576	0.235	2187.757	0.411	-6.117	0.246	-	0.051
7	YCYCRRRFCVCVGR	14	9.303	3.454	0.214	1784.163	0.5	74.278	-0.702	-	0.272
8	RGGRLCYCRRRFCVCT	16	9.88	4.456	0.125	1949.357	0.437	99.043	-0.452	-	0.283
9	KWKKFKKKLAGLLAKVLTT	19	10.778	6.539	0.105	2201.781	0.421	-7.37	0.044	-	0.116
10	KWARLWRWFRITRWLWYIK	19	11.999	5.549	0.368	2765.311	0.315	88.81	0.863	-	0.11
11	KKKTWWKTWTKWSQPKK	17	10.778	6.539	0.235	2275.694	0	5.547	-0.062	-	0.134

Further analysis using the attention mechanism embedded within the MOFormer model provides a nuanced, quantitative interpretation of the interactions between amino acid residues within the amphitropic regions of these peptides (see Supplementary Figure S2(b)). For example, the sequence represented by ID2, distinguished by its apex PIDDT score, exhibited pronounced attention between the arginine residues at positions R4 and R12. This suggests a potent interaction, likely mediated through hydrogen bonds, salt bridges, and other non-covalent interactions, contributing to the conformational integrity of the AMP sequence. Similarly, the interaction between glycine (G1) and arginine (R12) was noted, emphasizing the importance of electrostatic interactions in the structural integrity and folding dynamics of the peptide. These insights are crucial for understanding how AMPs engage with bacterial membranes and execute their antimicrobial mechanisms, offering potential avenues for the design of more effective antimicrobial agents.

To validate the superiority and universality of our meticulously designed MOFormer, we analyzed its performance relative to established methods such as LSTM, AMP-GAN, PepGAN, WAE, AMPEMO, and HMAMP, focusing on MIC and TOXI. As indicated in Table 3 (ID6-ID11), both MOFormer (w/o F) and MOFormer are highlighted as frontrunners, particularly MOFormer, which demonstrates a distinct advantage.

**Table 3:** Comparative performance metrics of AMP generation by MOFormer and other methods. This table displays the interval satisfaction proportions of AMPs generated by MOFormer and competing methods, focusing on $(MIC < 2)$ and $(TOXI < 0.5)$. It also presents the combined optimization scores and HV metrics, which evaluate the comprehensive performance of each method in achieving dual objectives of maximizing antimicrobial efficacy while minimizing TOXI

Method	MIC (<2)	TOXI (<0.5)	Combination	HV
LSTM	0.369	0.63	0.277	0.779
AMPEMO	0.749	0.842	0.657	0.69
PepGAN	0.777	0.945	0.743	1.081
WAE	0.617	0.881	0.565	1.06
AMP_GAN	0.646	0.984	0.64	0.946
HMAMP	0.722	0.956	0.706	1.154
MOFormer (w/o F)	0.731	0.923	0.696	1.138
MOFormer	0.745	0.936	0.717	1.291

While MOFormer and MOFormer (w/o F) significantly outperform other methods in combining MIC and TOXI properties, they exhibit a slight underperformance relative to PepGAN in certain aspects. However, this minor shortfall does not detract from the innovative nature of our methods. It is important to note that TOXI data are notoriously scarce and challenging to gather, which impacts the validation process for MOFormer and its variant.

Our analysis also shows that MOFormer excels in the HV metric, aligning perfectly with our goal to minimize the two-objective conflict within a bidimensional framework. This result underscores the superior and robust nature of our optimization strategy, highlighting MOFormer’s effectiveness in managing multiple objectives in AMP design.

To substantiate the viability of MOFormer, we replicated a rigorous screening mechanism aimed at identifying peptides with high antimicrobial activity and low TOXI potential. Initially, we analyzed sequence logos of 67 candidate peptides, as displayed in Supplementary Figure S4(a) and Table 2. Following this preliminary analysis, we narrowed our focus to six candidates, each comprising no more than 20 amino acids. We then conducted detailed structural sequence visualizations for these peptides (as shown in Supplementary Figure S3). IDs 6 through 11 predominantly revealed the presence of alpha-helices, with PIDDT scores ranging between 70-90%. These scores not only indicate high activity levels but also suggest considerable reliability in the structural predictions. We also constructed a detailed heatmap of amino acid interactions for ID8 to quantitatively analyze the inter-residue relationships, presented in Supplementary Figure S4(b).

As an illustrative case study, we assessed MOFormer’s ability to design AMPs addressing a tri-objective problem, focusing on balancing MIC, HEMO, and TOXI (see Supplementary Section D for details).

## Conclusion

In this study, we introduced MOFormer, a deep generative framework tailored for multi-objective AMP design. MOFormer significantly improves success rates over existing methods, though its performance on MIC and TOXI metrics is constrained by limited and noisy TOXI data. Leveraging a hierarchical candidate screening pipeline and fine-tuned proxy models, MOFormer efficiently ranks thousands of generated peptides, aiding in the selection of experimentally testable candidates. Despite challenges in validation, the generated AMPs exhibit desirable structural features—mainly $\alpha $-helicity and enrichment in cationic and hydrophobic residues—consistent with known AMP design principles. Our results demonstrate MOFormer’s capability in generating effective and realistic multi-property AMPs, as evidenced by *de novo* peptides with desirable multi-functional characteristics.

Despite these promising results, multi-objective optimization models—particularly conditional generative models—tend to converge toward a limited set of optimal regions when simultaneously minimizing MIC and TOXI. This behavior mirrors a form of mode collapse, wherein diversity is sacrificed to achieve precise control over target properties.

To overcome this limitation, future work will focus not only on enriching the availability of extreme-case training data, but also on advancing model architectures to more effectively preserve diversity under stringent constraints. One promising direction lies in the adoption of classifier-free diffusion models, which facilitate the seamless integration of both conditional and unconditional generative pathways within a unified framework. By jointly optimizing property-conditioned and unconditioned diffusion objectives, such models can simultaneously capture fine-grained, task-specific guidance and broader distributional priors, thereby enabling the modeling of complex, nonlocal spatial relationships that transcend simple sequence-level representations. This approach holds significant potential for enabling the generation of more robust, diverse, and well-balanced solutions, even under extreme multi-objective constraints.

In addition to these methodological advancements, subsequent work will also prioritize experimental validation of the generated peptide sequences. This will involve peptide synthesis and purification, followed by *in vitro* assays to assess MIC, HEMO, and TOXI. In parallel, molecular dynamics simulations will be employed to investigate membrane-binding mechanisms and conformational stability, providing structural and mechanistic insights. Ultimately, these efforts seek to bridge the gap between computational design and both *in vitro* and *in vivo* validation, facilitating the effective translation of multi-objective AMP generation into clinically relevant applications.

**Key Points**

- This study presents MOFormer, a novel multi-objective Transformer designed to optimize the trade-offs in conflicting multi-property design for antimicrobial peptides (AMPs). MOFormer leverages a structured latent space and advanced multi-objective optimization techniques to achieve superior performance in AMP design.
- MOFormer demonstrates excellent performance across diverse multi-objective spaces, achieving optimal HV and outperforming state-of-the-art methods. Its robustness and adaptability make it highly effective in various optimization scenarios.
- MOFormer enables robust screening through a non-dominated sorting algorithm, which generates Pareto decision curves and identifies top-ranked candidate solutions. Combined with fine-tuned surrogate models, it accelerates the discovery of potential AMP candidates.
- MOFormer enhances interpretability through multiple sequence alignment, molecular visualization, and attention score analysis. These tools provide detailed insights into sequence characteristics and structural functions, supporting further research and validation

## Supplementary Material

MoFormer_7_8-S_bbaf376

## Data Availability

The source code and processed datasets utilized in this study are publicly available at https://github.com/wl-wl/MOFormer/tree/master.
