# Supplementary material for: MOFormer: navigating the antimicrobial peptide design space with Pareto-based multi-objective transformer
Source: Brief Bioinform. 2025 Nov 5;26(6):bbaf376. doi: 10.1093/bib/bbaf376 (PMC12596111; doi:10.1093/bib/bbaf376)
Supplement: MoFormer_7_8-S_bbaf376 [file moformer_7_8-s_bbaf376.pdf]

MOFormer: Navigating the Antimicrobial Peptide Design Space with Pareto-based Multi-Objective Transformer

{Supporting Information}

Li Wang[1], Xiangzheng Fu[2], Jiahao Yang[3], Xinyi Zhang[3], Xiucai Ye[1,*], Tetsuya Sakurai[1], Xiangxiang Zeng[3], Yiping Liu[3,*]

[1] Department of Computer Science, University of Tsukuba, Tsukuba 305-8577, Japan
[2] School of Chinese Medicine, Hong Kong Baptist University, Hong Kong, China
[3] College of Computer Science and Electronic Engineering, Hunan University, Changsha, Hunan, China

*Corresponding Authors: Xiucai Ye (yexiucai@cs.tsukuba.ac.jp), Yiping Liu (yiping0liu@gmail.com)

A. Sequence Modeling

The objective of sequence modeling in our context, using the Transformer architecture, is to learn the probability distribution $p(x)$ of AMP sequences $x = (x_1, \ldots, x_n)$, composed of a finite set of amino acids. We use the chain rule of probability to factorize this distribution:

$$p(x) = \prod_{i=1}^{n} p(x_i | x_{<i}). \tag{1}$$

Training involves minimizing the negative log-likelihood of this distribution over our dataset $X = \{x^1, ..., x^n\}$:

$$\mathcal{L}(X) = -\sum_{k=1}^{n} \log p_\theta(x_i^k | x_{<i}^k). \tag{2}$$

A new AMP sequences of length n can be generated by iteratively sampling amino acids: $p_\theta(x_0), p_\theta(x_1|\tilde{x}_0), \ldots, p_\theta(x_n|\tilde{x}_{<n})$. After elementary preprocessing, each AMP sequence, comprising n tokens, is embedded as a sequence of n corresponding vectors. Each vector is the aggregate of a learned token embedding and a sinusoidal positional embedding as in the original Transformer architecture. The first core of the Transformer block is the multi-head attention with k heads that employs a causal masking strategy to prevent attending to future tokens:

$$\text{Attention}(Q, K, V) = \text{Softmax}\left(\frac{mask(QK^T)}{\sqrt{d}}\right) V, \tag{3}$$

where d is the dimension of K, and d must correspond to the dimension of Q.

$$\begin{aligned} \text{Multi-Head}(Q, K, V) &= \text{Concat}(\text{head}_1, \ldots, \text{head}_h) W_o, \\ \text{where} \quad \text{head}_i &= \text{Attention}(QW_i^Q, KW_i^K, VW_i^V), \end{aligned} \tag{4}$$

and i is the number of multi-heads, each head$_i$ calculates an attention score between Q and K from different viewpoints using the different weights (W_i^Q, W_i^K, W_i^V) belonging to each head. The second core block is the a feedforward network with ReLU activation, parameters W^F :

$$FF(X) = \max(0, XU) W^F. \tag{5}$$

Each block in the architecture commences with layer normalization, and follows it with a residual connection. In the first block, inputs X_i are first normalized, and then the normalized input $\bar{X}_i$ is processed through a multi-head attention mechanism which is added back to X_i via a residual connection.

$$\begin{aligned} \bar{X}_i &= \text{LayerNorm}(X_i), \\ H_i &= \text{MultiHead}(\bar{X}_i) + \bar{X}_i. \end{aligned} \tag{6}$$

The second block takes the output H_i from the first block, applies layer normalization, and then feeds it into a feed-forward network. The output from this network is added back to the normalized H_i to form another residual connection.

$$\begin{aligned} \bar{H}_i &= \text{LayerNorm}(H_i), \\ X_{i+1} &= \text{FF}(\bar{H}_i) + \bar{H}_i. \end{aligned} \tag{7}$$

During training, $X_{output} = (X_1, ..., X_{i+1})$ is the input of a cross-entropy loss function:

$$\mathcal{L}_{CE_{seq}}(X) = CossEntropyLoss(X_{output}, X'). \tag{8}$$

B. Condition Sampling

In this study, we focused on properties such as MIC, HEMO, TOXI and condensed descriptors. To analyze these, we constructed a five-dimensional histogram by dividing each property into 100 equal segments spanning from the minimum to the maximum values observed in the training dataset. Subsequently, histogram cells were populated based on the probability of occurrence of data samples within each cell. This probability was calculated as the ratio of the number of samples in a given cell to the total number of samples. Beam search [1] is a heuristic search algorithm widely implemented as the final decision-making layer in various natural language processing [6] and speech recognition models [2]. It determines the optimal output by sequentially selecting the top N predictions until an end-of-sequence marker is reached. In the inference phase, the decoder selects the next tokens iteratively through a 4-beam search and generates high-quality AMP sequences that conform to desired conditions.

C. Model Implementation Details

The MOFormer model adopts a symmetric encoder–decoder architecture, in which both the encoder and decoder are composed of six Pre-LayerNorm Transformer blocks. Each block is configured with a model dimensionality of 512, multi-head self-attention comprising eight heads (each with a dimensionality of 64), and a position-wise feed-forward network with a hidden size of 2048. A Gaussian latent space with 128 dimensions is utilized to capture compressed and informative representations of the input sequences.

We adopt a hybrid regularization strategy to mitigate posterior collapse in the discrete latent space, combining KL-weight annealing and Gaussian dropout. Specifically, a KL-weight (β) annealing schedule is applied, where β is initialized at 0.02 and linearly increased by 0.02 per epoch starting from epoch 1. The annealing continues until β reaches a maximum value of 1.0 after approximately 50 epochs. In parallel, we apply a fixed Gaussian dropout rate (α = 0.2) throughout the training process. This reparameterization-based dropout introduces stochasticity in the latent variables, enhancing robustness without any adaptive tuning of α. For optimization, we utilize the Adam optimizer (β_1 = 0.9, β_2 = 0.98, $\theta = 1 \times 10^{-9}$) with an initial learning rate of 0.0001. A linear warm-up schedule comprising 8000 steps is employed before transitioning into steady-state learning. To ensure reproducibility, we fix the random seed to 123.

Furthermore, the model integrates fully connected layers with a hidden dimensionality of 512 for sequence score prediction, applies a dropout rate of 0.3 to mitigate overfitting and promote generalization, and was trained over 25 epochs with a batch size of 1024. All computational experiments were conducted using the MOFormer framework implemented in PyTorch on a single NVIDIA A100-PCIE-40GB GPU within approximately 2 to 2.5 hours. This configuration underscores the model's efficiency and the feasibility of implementing advanced deep learning techniques in a relatively constrained time frame.

D. Optimization of Existing AMPs Towards Triple Objectives

As an illustrative case study, we assessed MOFormer's ability to design AMPs addressing a tri-objective problem, focusing on balancing MIC, HEMO, and TOXI. Specifically, this involves minimizing MIC values, HEMO probability, and TOXI probability at the same time. We expanded the conditions in Equations 11 to ensure that the MOFormer encoder comprehensively captures the likelihood distributions of the target conditions, facilitating the generation of diverse AMPs candidates under these guided conditions. Additionally, we refined the latent space through Equation 16 and introduced a loss function enriched with conditional information to optimize the model. This optimization allows for the generation of peptides that meet the desired specifications under specified conditions. We utilized a large-model-based fine-tuning predictor to assess the properties of these candidates and applied non-dominated sorting to construct a three-dimensional Pareto frontier. Detailed descriptions are provided in the Methods.

In our experiments, we utilized HV as the evaluation metric with a reference point set at (2,0.5,0.5), corresponding to our targets for antimicrobial activity ($log(MIC) < 2$), HEMO probability ($HEMO < 0.5$) and TOXI probability ($TOXI < 0.5$). The performance of MOFormer on the tri-objective task was benchmarked against six state-of-the-art methods: LSTM [5], AMP-GAN [3], PepGAN [7], WAE [3], AMPEMO [4], and HMAMP [8]. As illustrated in Figure **S5**(a), MOFormer achieves higher HV values compared to other advanced methods, indicating superior diversity and quality in the solution sets, and suggesting the discovery of a greater number of more optimal solutions. Furthermore, Supplementary Figure S5(b) shows that the solutions are distinctly clustered around low MIC, low HEMO, and low TOXI values. Although some solutions fall short of expectations concerning the HEMO attribute, the inherent conflicting properties of AMPs cannot be overlooked. In summary, MOFormer offers an effective approach for the designing and screening of AMPs with triple objectives.

TABLE I
NOVELTY OF MOFORMER-GENERATED SEQUENCES VS. TRAINING DATA ACROSS DIFFERENT THRESHOLDS

Threshold(T)	Novelty Proportion(%)
1	38.60
2	33.10
3	28.90
4	24.69
5	22.30

TABLE II
T-TEST STATISTICS FOR COMPARATIVE ANALYSIS OF AMPS. THIS TABLE PRESENTS THE P-VALUES FROM T-TESTS CONDUCTED TO COMPARE THE PERFORMANCE OF AMPS GENERATED BY MOFORMER, ITS ABLATION VARIANTS, AND THE BENCHMARK DATASETS (TRAIN AND TEST). THE TESTS ASSESS THE SIGNIFICANCE OF DIFFERENCES IN THE MULTI-ATTRIBUTE PERFORMANCE (MIC AND HEMO) OF AMPS GENERATED BY THE FULL MOFORMER MODEL VERSUS THOSE GENERATED IN TRAINING, TESTING, MOFORMER (W/O D), AND MOFORMER (W/O F) SCENARIOS.

T-test	MOFormer			
	Train Datasets	Test Datasets	MOFormer(w/o D)	MOFormer(w/o F)
MIC	0.0234	0.381	0.0006	0.595
HEMO	0.0462	0.343	0.3307	0.854

- S1. Visualization of the Latent Encoding Space and Attribute Landscapes.
- S2. A comprehensive view of the antimicrobial activity and HEMO of candidate peptides generated by MOFormer.
- S3. A comprehensive view of the antimicrobial activity and TOXI of candidate peptides generated by MOFormer.
- S4. Displays the molecular visualization of AMP structures for candidates ID6 through ID11

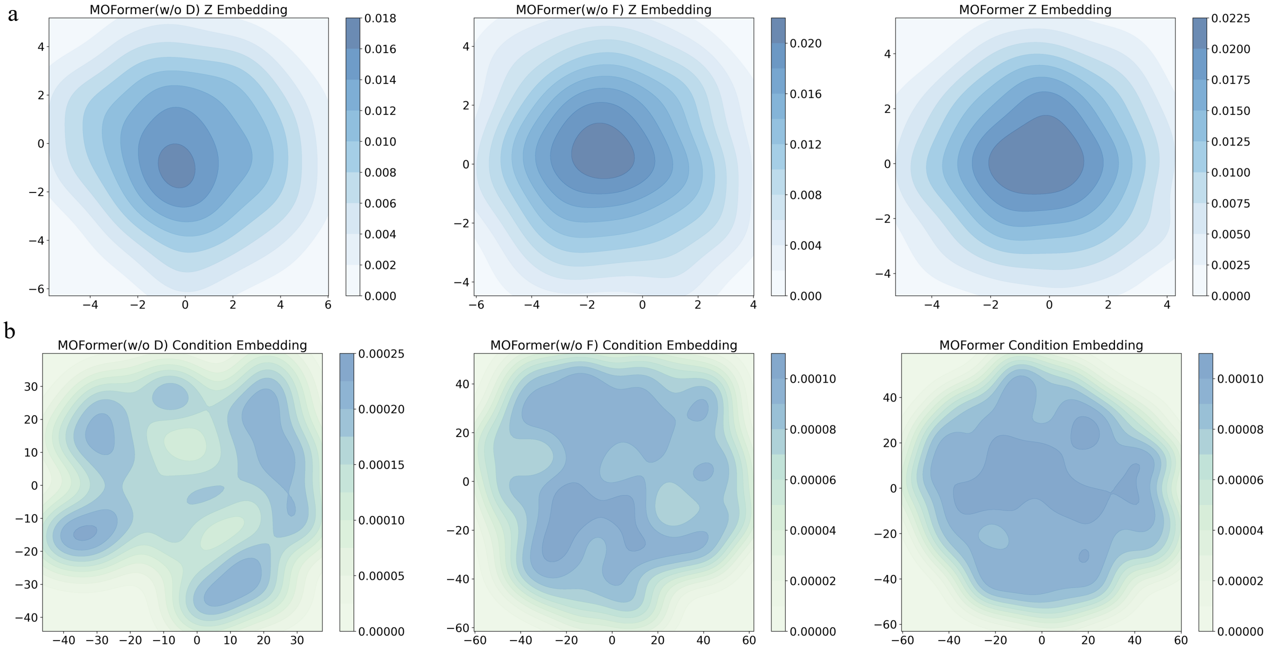

Figure S1. Visualization of the Latent Encoding Space and Attribute Landscapes. This figure illustrates the encoding space Z and the condition landscape for three configurations of MOFormer: MOFormer (w/o D), MOFormer (w/o F), and full MOFormer. By mapping these landscapes, we demonstrate how each variant explores and occupies the latent space, offering insights into how property constraints and optimization objectives are navigated and achieved within this space. The visualization helps in understanding the differential impact of design choices on the efficacy and specificity of the generated AMPs.

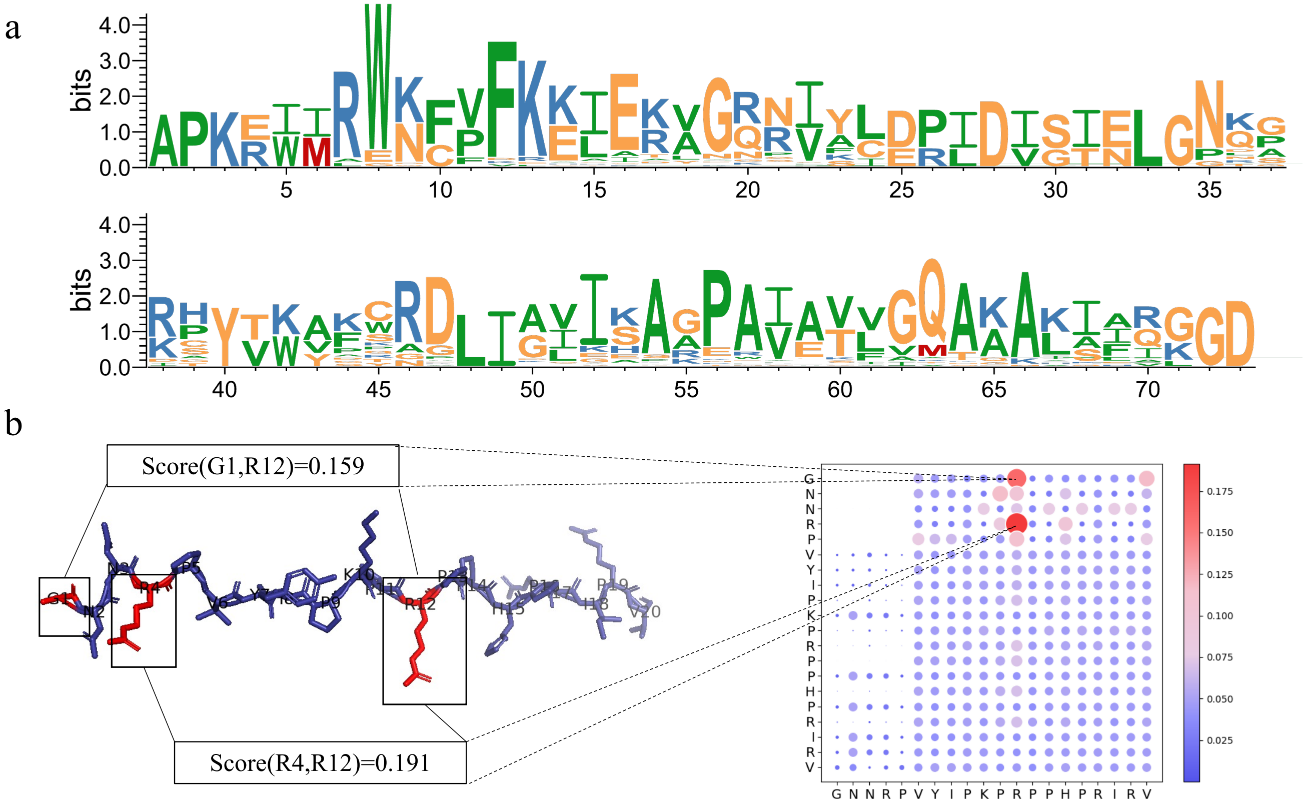

Figure S2. A comprehensive view of the antimicrobial activity and HEMO of candidate peptides generated by MOFormer. a. Sequence logos of candidate peptides generated by MOFormer, illustrating the conservation and variability of amino acids across selected peptides, which informs their functional potential in antimicrobial activities. b. Attention weights extracted from the transformer module, offering deeper insights into the interpretation of AMP structure and function. This component highlights the model's ability to focus on critical regions of the peptide sequence that are pivotal for antimicrobial activity and HEMO.

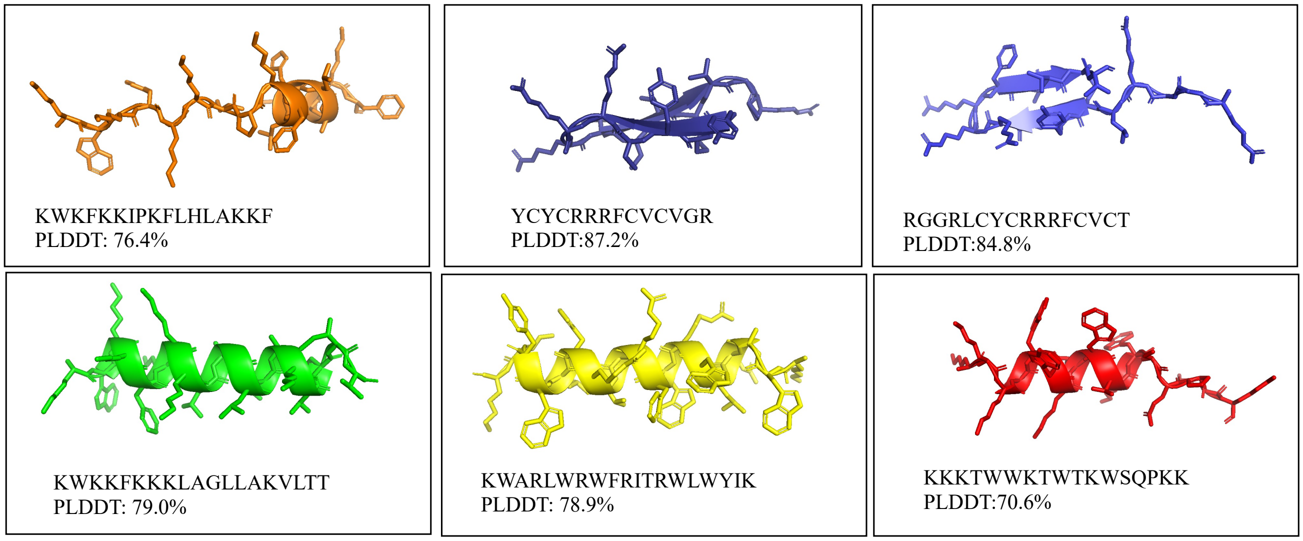

Figure S3. Displays the molecular visualization of AMP structures for candidates ID6 through ID11(in table 2), showing the three-dimensional conformation crucial for their bioactivity.

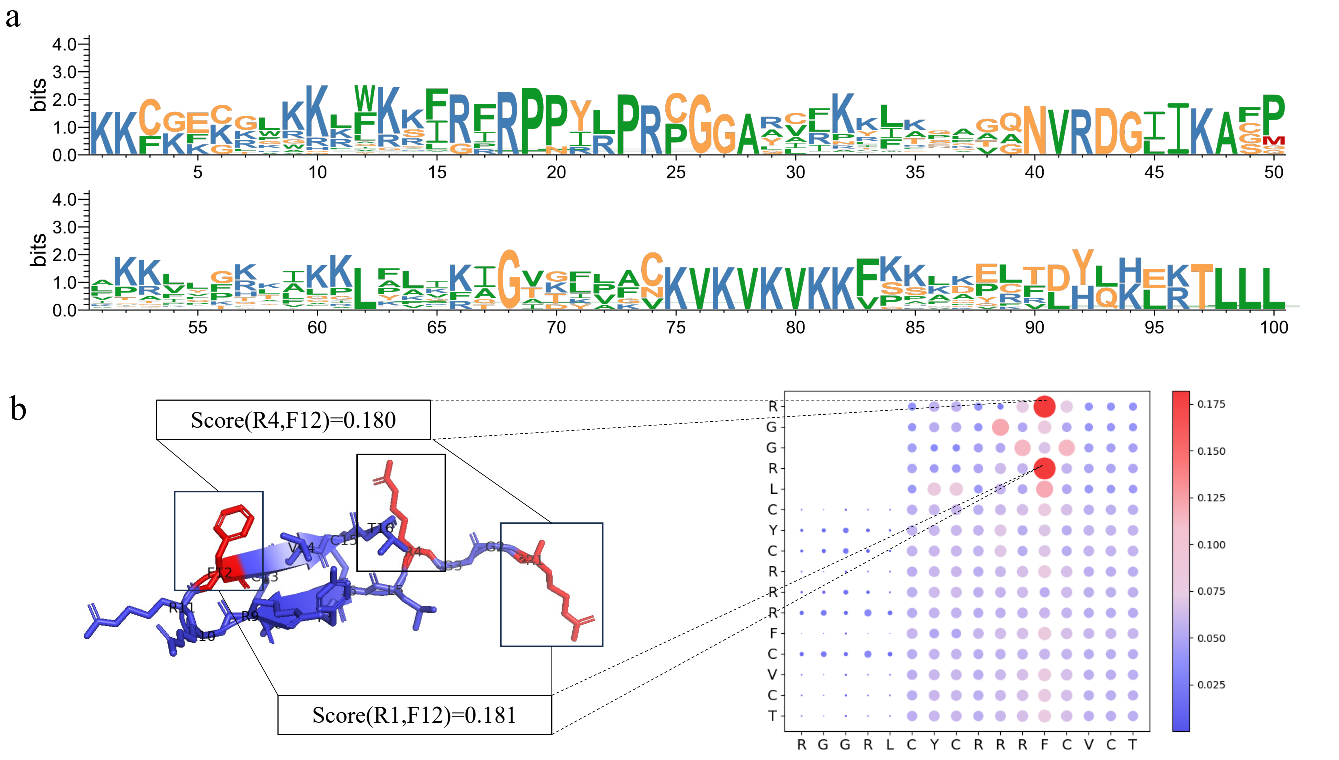

Figure S4. A comprehensive view of the antimicrobial activity and TOXI of candidate peptides generated by MOFormer. a. Sequence Logos: Illustrates the sequence logos of candidate peptides, highlighting the conservation and variability of amino acids at specific positions, which is crucial for understanding the peptides' functional characteristics. b. Attention Weights: Extracted from the transformer module, these weights offer deeper insights into the AMPs' structure and function by identifying key residues and interactions that contribute to their efficacy and specificity.

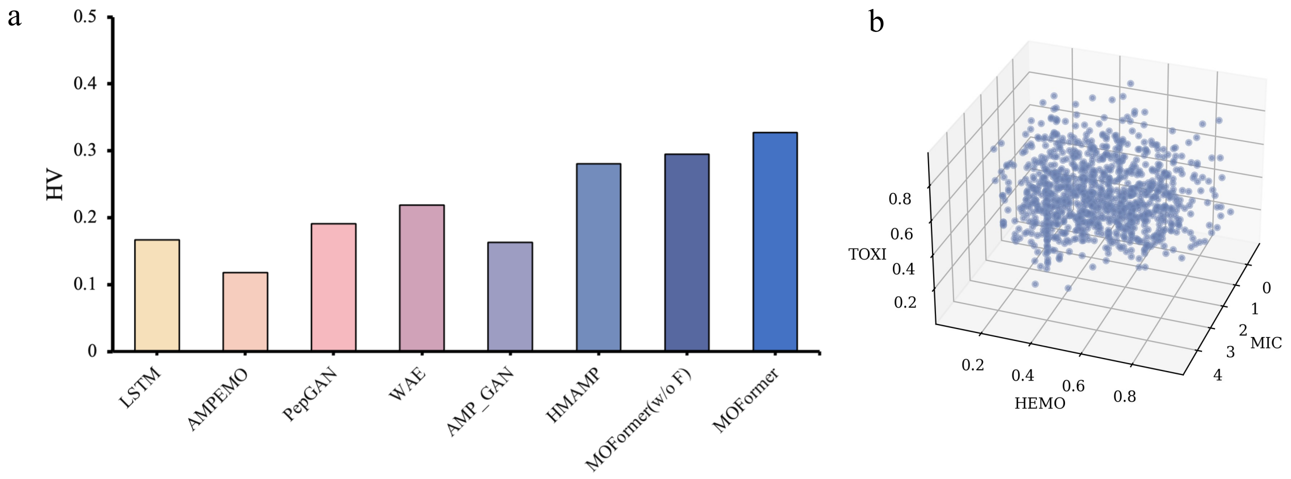

Figure S5. a. HV comparison results of MOFormer and other state-of-the-art methods on the triple-objective scenarios. b. Visualization of MOFormer solution set.

References

[1] Markus Freitag and Yaser Al-Onaizan. Beam search strategies for neural machine translation. *arXiv preprint arXiv:1702.01806*, 2017.

[2] Alex Graves, Abdel-rahman Mohamed, and Geoffrey Hinton. Speech recognition with deep recurrent neural networks. In *2013 IEEE international conference on acoustics, speech and signal processing*, pages 6645–6649. Ieee, 2013.

[3] Tzu-Tang Lin, Li-Yen Yang, Ching-Tien Wang, Ga-Wen Lai, Chi-Fong Ko, Yang-Hsin Shih, Shu-Hwa Chen, and Chung-Yen Lin. Discovering novel antimicrobial peptides in generative adversarial network. *BioRxiv*, pages 2021–11, 2021.

[4] Yiping Liu, Xinyi Zhang, Yuansheng Liu, Yansen Su, Xiangxiang Zeng, and Gary G Yen. Evolutionary multi-objective optimization in searching for various antimicrobial peptides [feature]. *IEEE Computational Intelligence Magazine*, 18(2):31–45, 2023.

[5] Alex T Muller, Jan A Hiss, and Gisbert Schneider. Recurrent neural network model for constructive peptide design. *Journal of chemical information and modeling*, 58(2):472–479, 2018.

[6] Ilya Sutskever, Oriol Vinyals, and Quoc V Le. Sequence to sequence learning with neural networks. *Advances in neural information processing systems*, 27, 2014.

[7] Andrejs Tucs, Duy Phuoc Tran, Akiko Yumoto, Yoshihiro Ito, Takanori Uzawa, and Koji Tsuda. Generating ampicillin-level antimicrobial peptides with activity-aware generative adversarial networks. *ACS omega*, 5(36):22847–22851, 2020.

[8] Li Wang, Yiping Liu, Xiangzheng Fu, Xiucai Ye, Junfeng Shi, Gary G Yen, Quan Zou, Xiangxiang Zeng, and Dongsheng Cao. Hmamp: Designing highly potent antimicrobial peptides using a hypervolume-driven multiobjective deep generative model. *Journal of Medicinal Chemistry*, 68(8):8346–8360, 2025.
